# Characterization of Immune Responses Induced by Immunization with the HA DNA Vaccines of Two Antigenically Distinctive H5N1 HPAIV Isolates

**DOI:** 10.1371/journal.pone.0041332

**Published:** 2012-07-31

**Authors:** Yulong Gao, Zhiyuan Wen, Ke Dong, Gongxun Zhong, Xiaomei Wang, Zhigao Bu, Hualan Chen, Ling Ye, Chinglai Yang

**Affiliations:** 1 National Key Laboratory of Veterinary Biotechnology, Harbin Veterinary Research Institute, Chinese Academy of Agricultural Sciences, Harbin, People’s Republic of China; 2 Department of Microbiology and Immunology and Emory Vaccine Center, Emory University School of Medicine, Atlanta, Georgia, United States of America; 3 Central Laboratory, Tangdu Hospital, Fourth Military Medical University, Xi’an, People’s Republic of China; Erasmus Medical Center, Netherlands

## Abstract

The evolution of the H5N1 highly pathogenic avian influenza virus (HPAIV) has resulted in high sequence variations and diverse antigenic properties in circulating viral isolates. We investigated immune responses induced by HA DNA vaccines of two contemporary H5N1 HPAIV isolates, A/bar-headed goose/Qinghai/3/2005 (QH) and A/chicken/Shanxi/2/2006 (SX) respectively, against the homologous as well as the heterologous virus isolate for comparison. Characterization of antibody responses induced by immunization with QH-HA and SX-HA DNA vaccines showed that the two isolates are antigenically distinctive. Interestingly, after immunization with the QH-HA DNA vaccine, subsequent boosting with the SX-HA DNA vaccine significantly augmented antibody responses against the QH isolate but only induced low levels of antibody responses against the SX isolate. Conversely, after immunization with the SX-HA DNA vaccine, subsequent boosting with the QH-HA DNA vaccine significantly augmented antibody responses against the SX isolate but only induced low levels of antibody responses against the QH isolate. In contrast to the antibody responses, cross-reactive T cell responses are readily detected between these two isolates at similar levels. These results indicate the existence of original antigenic sin (OAS) between concurrently circulating H5N1 HPAIV strains, which may need to be taken into consideration in vaccine development against the potential H5N1 HPAIV pandemic.

## Introduction

Influenza virus infection causes serious respiratory illness, and seasonal human influenza epidemics are estimated to result in about 40,000 deaths and over 200,000 hospitalizations annually in the U.S. alone and up to 1.5 million deaths worldwide [1], [2]. Influenza virus contains a segmented negative-strand RNA genome and are categorized into three different types (A, B, and C) based on the antigenic properties of its two internal proteins, nucleoprotein and matrix protein [3]. Types B and C influenza viruses are primarily human pathogens, whereas the type A influenza virus exists in both humans and a number of animal species and has a natural reservoir in aquatic birds. Influenza A viruses are further divided into different subtypes based on their surface glycoproteins, hemagglutinin (HA) and neuraminidase (NA). To date, 16 subtypes of HA (H1–H16) and 9 subtypes of NA (N1–N9) glycoproteins have been identified in influenza A viruses. However, only 3 HA subtypes (H1–H3) and 2 NA subtypes (N1–N2) have been circulated and caused pandemic and seasonal influenza epidemics. Historically, three influenza A pandemics have occurred in the last century with the appearance of each new HA subtype [4]. In March and April of 2009, outbreaks of a new H1N1 influenza virus in humans emerged in California of the United States and in Mexico, which subsequently spread worldwide and led to the declaration of a new influenza pandemic by WHO in June 2009 [5]. Characterization of the new H1N1 influenza virus showed that it is of swine origin [6], [7]. The rapid spread of the new H1N1 influenza virus demonstrates that a new human influenza pandemic of zoonotic origin poses a real threat to the public health.

Several subtypes of avian influenza virus have also been postulated to possess pandemic potential and the H5N1 highly pathogenic avian influenza virus (HPAIV) is of particular concern [8]. The first human outbreak of H5N1 HPAIV occurred in 1997 in Hongkong, China as a result of direct avian-to-human transmission that led to 18 human infections with 6 deaths [9], [10]. While massive culling of poultry effectively controlled human outbreak for several years, H5N1 HPAIV remained endemic in poultry species in Southern China [11]–[13]. In late 2003, new human outbreaks of H5N1 HPAIV occurred in the Southeast Asia [14], and the virus has since then spread to Europe and Africa, causing over 600 human infections with 356 deaths as of May, 2012 according to the World Health Organization [15]. The ability of H5N1 HPAIV to directly infect humans with a high fatality rate (almost 60%) makes it a great threat as a causative agent for a potential new influenza pandemic. Moreover, the evolution of H5N1 HPAIV in wild birds and farm poultry has resulted in concurrent circulation of diverse virus strains with distinctive antigenic properties [16], and H5N1 HPAIV of different antigenic lineages has been reported to cause direct infection in humans [17]. The high sequence variation in circulating H5N1 HPAIV poses a great challenge for the development of a vaccine strategy for the control of a potential H5N1 pandemic.

Based on genetic analysis of the HA gene, H5N1 HPAIV isolates have been categorized into 10 different clades that exhibit different antigenic properties [18]. However, information on cross reactivity of antibody responses between antigenically different H5N1 HPAIV isolates is still lacking. In this study, we investigated immune responses induced by HA DNA vaccines of two H5N1 HPAIV isolates, A/bar-headed goose/Qinghai/3/2005 (QH) and A/chicken/Shanxi/2/2006 (SX), that are representatives of two HPAIV antigenic lineages clade 2.2 and clade 7 respectively [19]. The QH virus was isolated in an outbreak in the Qinghai Lake of China in 2005 that caused massive deaths of wild birds [20], whereas the SX virus was isolated in an outbreak in Shanxi, China in farm poultry in 2006 [21]. Both viruses are highly pathogenic to domestic chickens and the HA proteins contain a polybasic amino acid segment that is characteristic of H5N1 HPAIV. Their HA protein sequence differs by about 7% and we recently reported that these two viruses do not exhibit significant cross reactivity in chicken [19]. In this study, we further investigated the immunogenicities of the QH and SX HA DNA vaccines in mice. Our results show that the HA of these two viruses are antigenically distinctive in mice. Interestingly, by carrying out heterologous priming-boosting immunizations, we observed that boosting with a heterologous HA effectively augmented antibody responses to the HA of the priming virus strain but not to the HA of the boosting virus strain, indicating the existence of original antigenic sin (OAS) between these two concurrently circulating H5N1 HPAIV strains.

## Results

### Immunization with the QH-HA and SX-HA DNA Vaccines Induces Strong Antibody Responses Against the Homologous Strain but not to the Heterologous Strain

**Figure 1:**
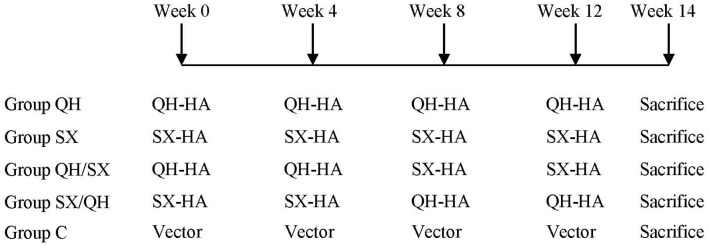
Schematic diagram of immunization study design. Five groups of mice (groups of 6) were immunized with different regimens of QH and/or SX H5N1 HA DNA vaccines as indicated. Group QH and SX were immunized four times with the same HA DNA vaccine. Groups QH/SX and SX/QH were first immunized two times with the HA DNA vaccine of one virus strain and then boosted two times with the HA DNA vaccine of the other virus strain. Group C were immunized with control plasmid vector pCAGGS. All immunizations were carried out by intramuscular injection of 50 µg DNA vaccines at 4-week intervals. Blood samples were collected at two weeks after each immunization and were designated by the number (1, 2, 3, or 4) following the designated name of each group as used in subsequent figures. Mice were sacrificed at two-weeks after the final immunization to prepare splenocytes for analysis of T cell responses.

**Figure 2:**
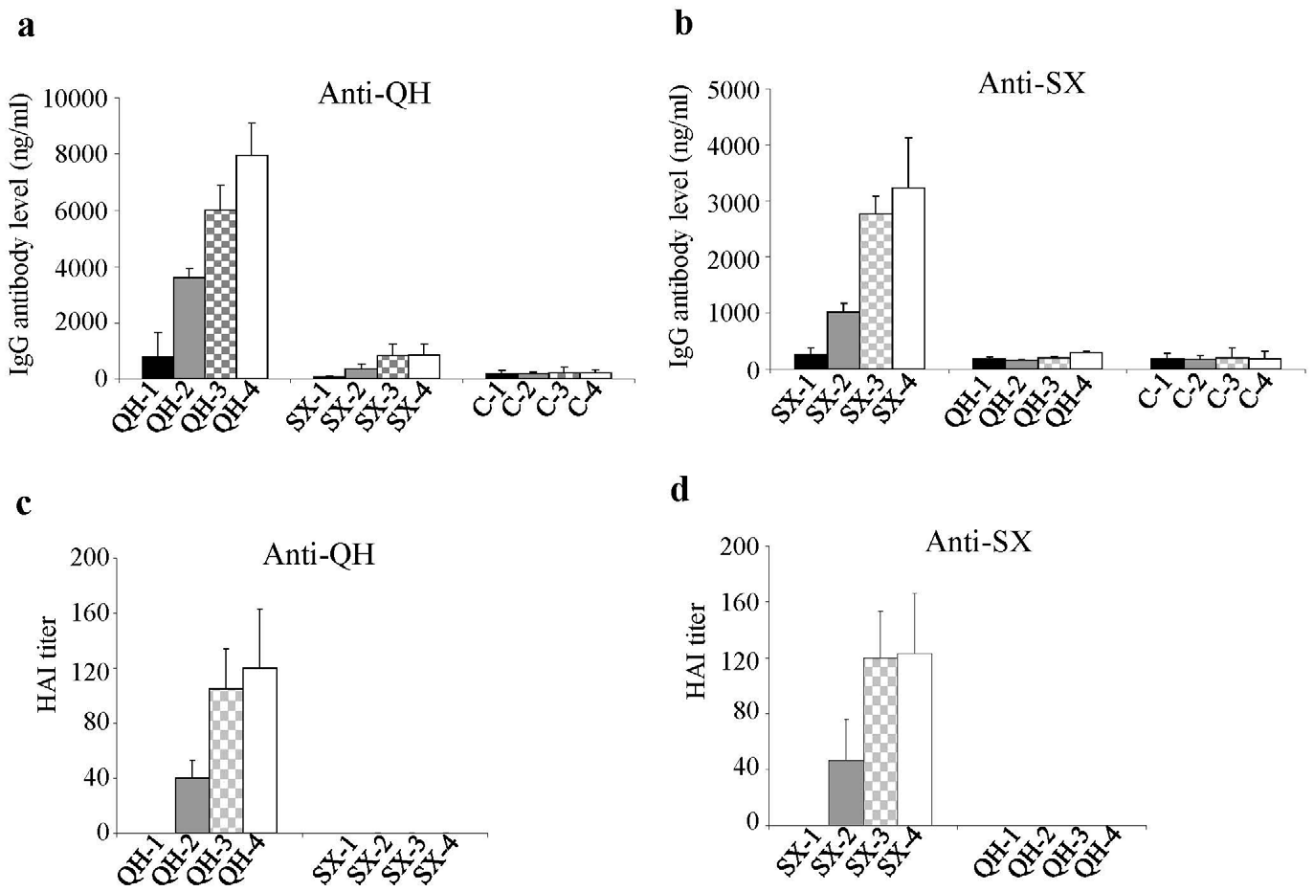
Characterization of antibody responses induced by QH and SX H5N1 HA DNA vaccines. Blood samples from mice vaccinated with QH (Group QH) or SX (Group SX) HA DNA vaccines were collected after each immunization and analyzed for antibody responses against homologous as well as heterologous viruses. The levels of antibody responses in serum samples were determined by ELISA using purified inactivated QH or SX virus as coating antigens as indicated, and expressed as the amount of virus-specific antibodies in 1 ml of serum samples (ng/ml). The HAI activity (HAI titer) was determined as the highest serum dilution that resulted in complete inhibition of hemagglutination by inactivated QH or SX virus as indicated. (**a**) Antibody responses against the QH virus. (**b**) Antibody responses against the SX virus. (**c**) HAI activity against the QH virus. (**d**) HAI activity against the SX virus. Data are presented as the mean ± standard deviation. Dashed lines indicate detection limit for HAI titer (1∶20).

We constructed DNA vaccines expressing the HA of the QH and SX H5N1 influenza viruses and characterized their expression in HeLa cells by transfection and Western blot, which showed these two HA proteins were expressed at similar levels (Figure S1). We further evaluated their immunogenicity and cross-reactivity in mice as outlined in Figure 1. Blood samples were collected at two weeks after each immunization and analyzed for antibody responses against the QH and SX viruses, and a summary of the antibody responses after each immunization is provided in Table S1. As shown in Figure 2a, immunization with the QH-HA DNA vaccine induced significant levels of antibody responses against the QH virus after two immunizations as compared to the control group (p<0.05). The third immunization further boosted the antibody levels significantly, whereas the fourth immunization only moderately increased antibody levels against the QH virus. In contrast, even after the fourth immunization with the QH-HA DNA vaccine, no significant level of antibody response against the SX virus was induced as compared to the control group (Figure 2b. QH-4 vs. C-4 against SX virus. p>0.1). Similarly, immunization by SX-HA DNA vaccine induced strong antibody responses against the SX virus (Figure 2b) but not to the QH virus (Figure 2a. SX-4 vs. C-4 against QH virus. p>0.1). In agreement with the results from ELISA studies, antibodies induced by QH-HA DNA vaccine exhibit hemagglutination inhibition (HAI) activity against the QH virus (Figure 2c) but not the SX virus (Figure 2d), whereas antibodies induced by the SX-HA DNA vaccine exhibit HAI activity against the SX virus (Figure 2d) but not the QH virus (Figure 2c). These results show that, similar as observed in chickens, the QH and SX HA exhibit distinctive antigenic properties in mice.

### A Single Boost with a Heterologous HA DNA Vaccine Augments Antibody Responses against the Original Virus Strain

**Figure 3:**
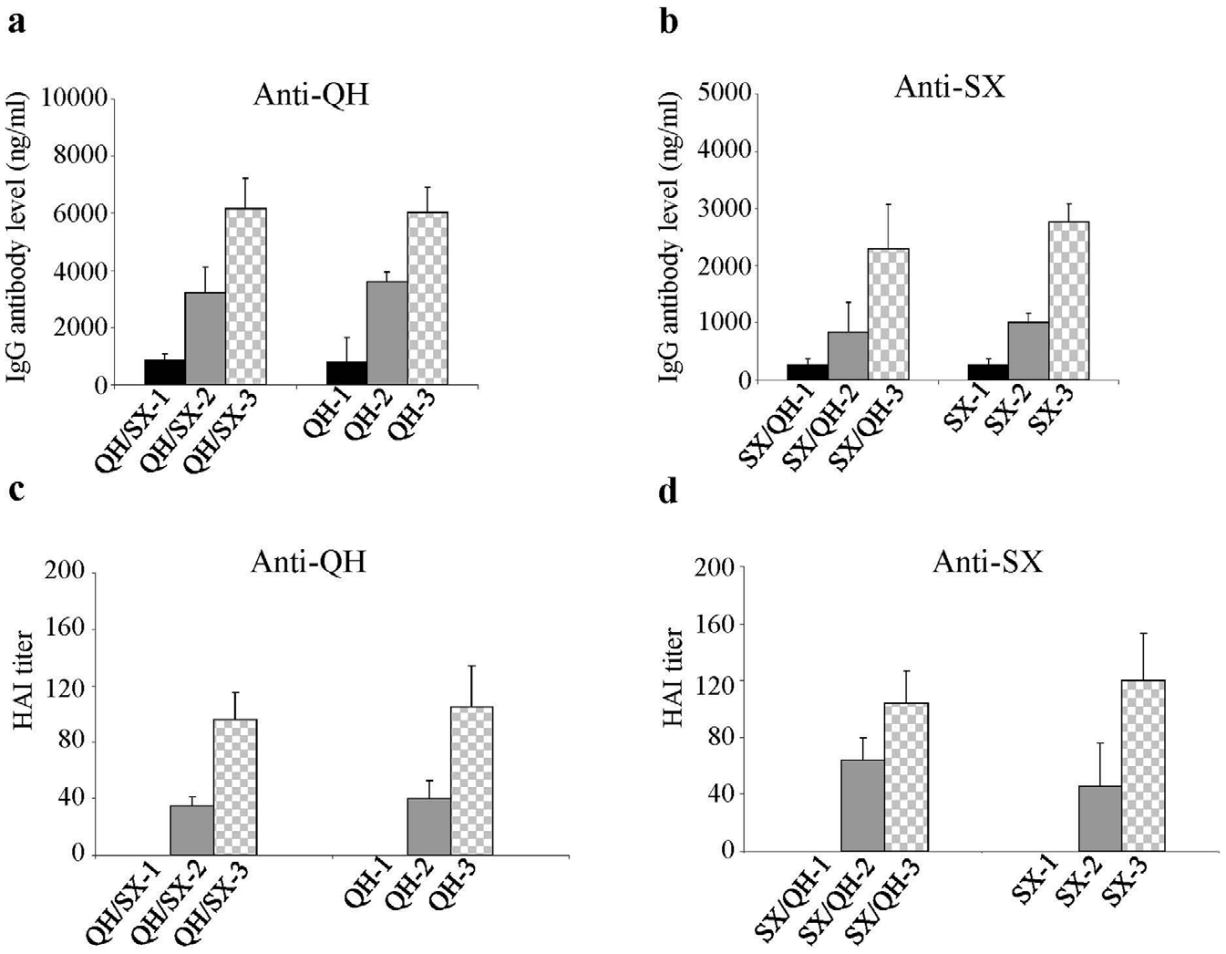
Antibody responses against the primary virus train were effectively boosted by a single heterologous HA DNA immunization. Blood samples collected from Groups QH/SX and SX/QH after the first heterologous boost immunization were analyzed for antibody responses against the primary virus strain, QH or SX viruses respectively, from which the HA DNA vaccine was used in priming immunizations. The results were compared with Groups QH or SX respectively that received homologous boosting immunizations. (**a**) Antibody responses against the QH virus. (**b**) Antibody responses against the SX virus. (**c**) HAI activity against the QH virus. (**d**) HAI activity against the SX virus. Data are presented as the mean ± standard deviation. Dashed lines indicate detection limit for HAI titer (1∶20).

**Figure 4:**
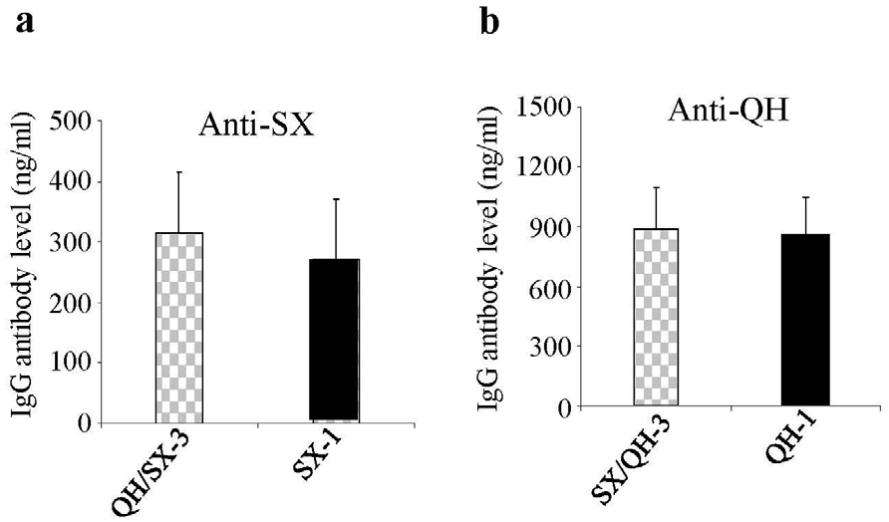
A heterologous HA DNA boosting induced similar levels of antibodies against the second virus strain as a single immunization with the HA DNA vaccine of the second strain. Blood samples collected from Groups QH/SX and SX/QH after the first heterologous boost immunization were analyzed for antibody responses against the second virus strain, SX or QH viruses respectively, from which the HA DNA vaccine was used in boosting immunizations. The results were compared with Groups SX or QH respectively after a single immunization. (**a**) Antibody responses against the SX virus. (**b**) Antibody responses against the QH virus. Data are presented as the mean ± standard deviation.

To further investigate the cross-reactivity of these two HA antigens, we also characterized the antibody responses induced in mice that received boosting immunizations with a heterologous HA DNA vaccine as outlined in Figure 1. As shown in Figure 3a, a single boosting immunization with the SX-HA DNA vaccine following two immunizations with the QH-HA DNA vaccine significantly augmented antibody responses against the QH virus (p<0.05, QH/SX-3 vs. QH/SX-2 against QH virus), to a similar level as those induced by three homologous immunizations with the QH-HA DNA vaccine (comparing QH/SX-3 and QH-3). Conversely, as shown in Figure 3b, a boosting immunization with the QH-HA DNA vaccine following two immunizations with the SX-HA DNA vaccine augmented antibody responses against the SX virus to a similar level as those induced by three immunizations with the SX-HA DNA vaccine (comparing SX/QH-3 and SX-3). Functional analysis of the antibody responses showed that the HAI activity of antibodies against the priming virus strain was also increased after a boosting immunization with the heterologous HA DNA vaccine (Figure 3c and 3d). On the other hand, in mice that received two QH-HA DNA immunizations, boosting with the SX-HA DNA vaccine induced similar levels of antibody responses against the SX virus to those induced by a single immunization with the SX-HA DNA vaccine (Figure 4a, comparing QH/SX-3 and SX-1). Similarly, in mice that received two SX-HA DNA immunizations, boosting with the QH-HA DNA vaccine induced similar levels of antibody responses against the QH virus to those induced by a single immunization with the QH-HA DNA vaccine (Figure 4b, comparing SX/QH-3 and QH-1). These results show that boosting by a heterologous HA DNA vaccine effectively augmented antibody responses to the priming virus strain. However, such antibodies were not reactive to the boosting virus strain.

### A Second Boost with the Heterologous HA DNA Vaccine only Augmented Antibody Responses against the Second Virus Strain

**Figure 5:**
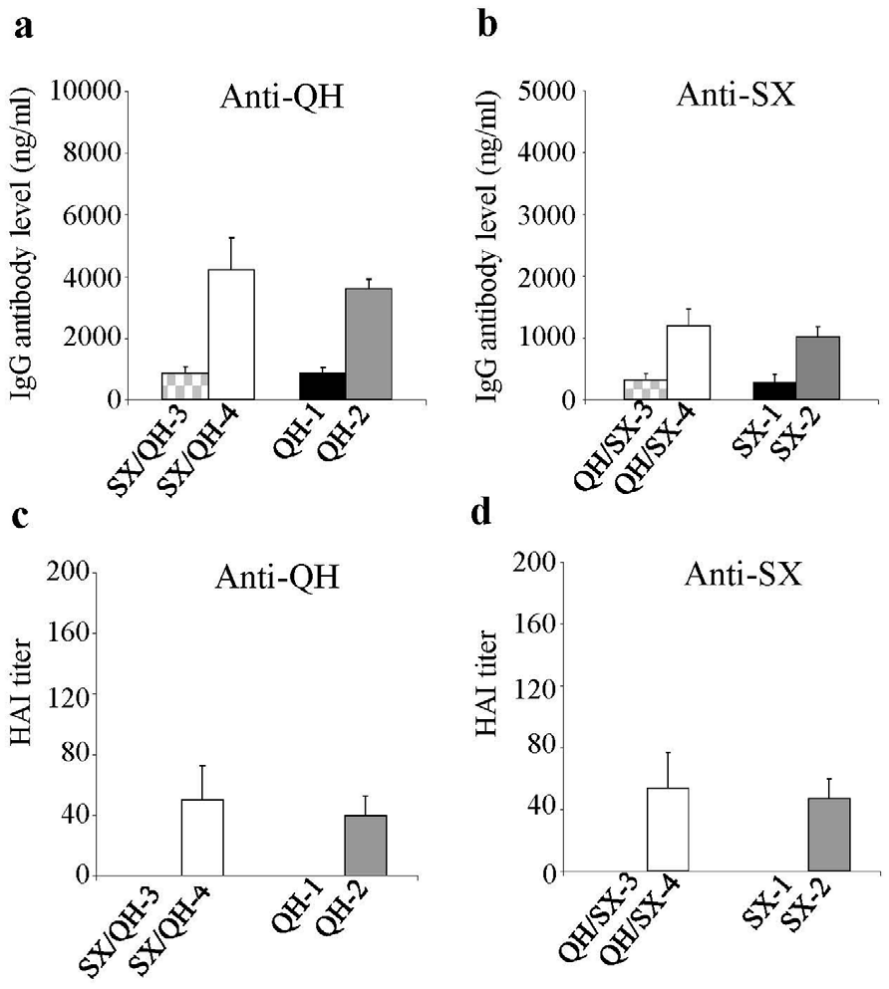
A second boost with the heterologous HA DNA vaccine augmented antibody responses against the second virus strain. Blood samples collected from Groups QH/SX and SX/QH after the second heterologous boost immunization were analyzed for antibody responses against the second virus strain (QH or SX virus respectively) in comparison with sera collected from Groups SX or QH respectively after two immunizations with the SX or QH HA DNA vaccines. (**a**) Antibody responses against the QH virus. (**b**) Antibody responses against the SX virus. (**c**) HAI activity against the QH virus. (**d**) HAI activity against the SX virus. Data are presented as the mean ± standard deviation. Dashed lines indicate detection limit for HAI titer (1∶20).

**Figure 6:**
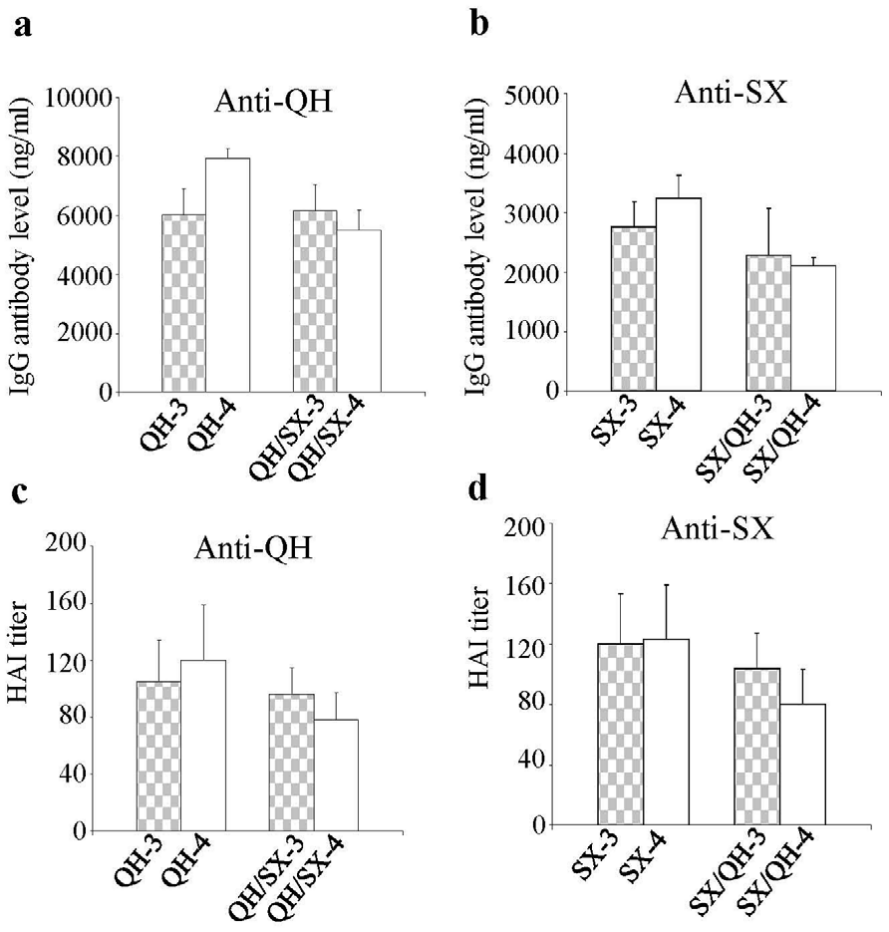
Antibody responses against the original virus strain were not enhanced by the second boost with a heterologous HA DNA vaccine. Blood samples collected from Groups QH/SX and SX/QH after the first and second heterologous boost immunization were analyzed for antibody responses against the primary virus strain (QH or SX virus respectively) from which the HA DNA vaccine was used in priming immunizations, in comparison with sera collected from Groups QH or SX respectively after the third and fourth immunizations with the HA DNA vaccine of the primary virus strain. (**a**) Antibody responses against the QH virus. (**b**) Antibody responses against the SX virus. (**c**) HAI activity against the QH virus. (**d**) HAI activity against the SX virus. Data are presented as the mean ± standard deviation. Dashed lines indicate detection limit for HAI titer (1∶20).

To investigate the impact of pre-existing antibody responses to the HA of the priming virus strain on antibody induction to the HA of the boosting virus strain, we further analyzed antibody responses against HA of both priming and boosting virus strains after a second boosting immunization to the mice with the heterologous HA DNA vaccine. As shown in Figures 5a and 5c, after the second heterologous boost with the QH HA DNA vaccine, antibody responses as well as HAI activity against the QH virus in Group SX/QH were boosted to similar levels as those induced by two immunizations with the QH HA DNA vaccine (comparing SX/QH-4 and QH-2 against the QH virus). On the other hand, as shown in Figure 6a and 6c, antibody levels as well as HAI titer against the SX virus (the priming virus strain) dropped slightly after the second boosting immunization with the QH HA DNA vaccine (comparing SX/QH-4 and SX/QH-3 against the SX virus). Similarly, the second boost with the SX HA DNA vaccine augmented antibody responses and HAI activity against the SX virus in Group QH/SX to similar levels as those induced by two immunizations with the SX HA DNA vaccine (Figures 5b and 5d. comparing QH/SX-4 and SX-2 against SX virus), while antibody responses against the QH virus (the priming virus strain) slightly dropped (Figure 6b and 6d). These results show that the second boosting immunization with the heterologous HA DNA vaccine effectively augmented the antibody responses as well as the HAI activity against the boosting virus strain but not the priming virus strain.

### The T Cell Responses Induced by Different Immunization Regimens with QH and SX HA DNA Vaccines are at Similar Levels and Exhibit Cross-reactivity

**Figure 7:**
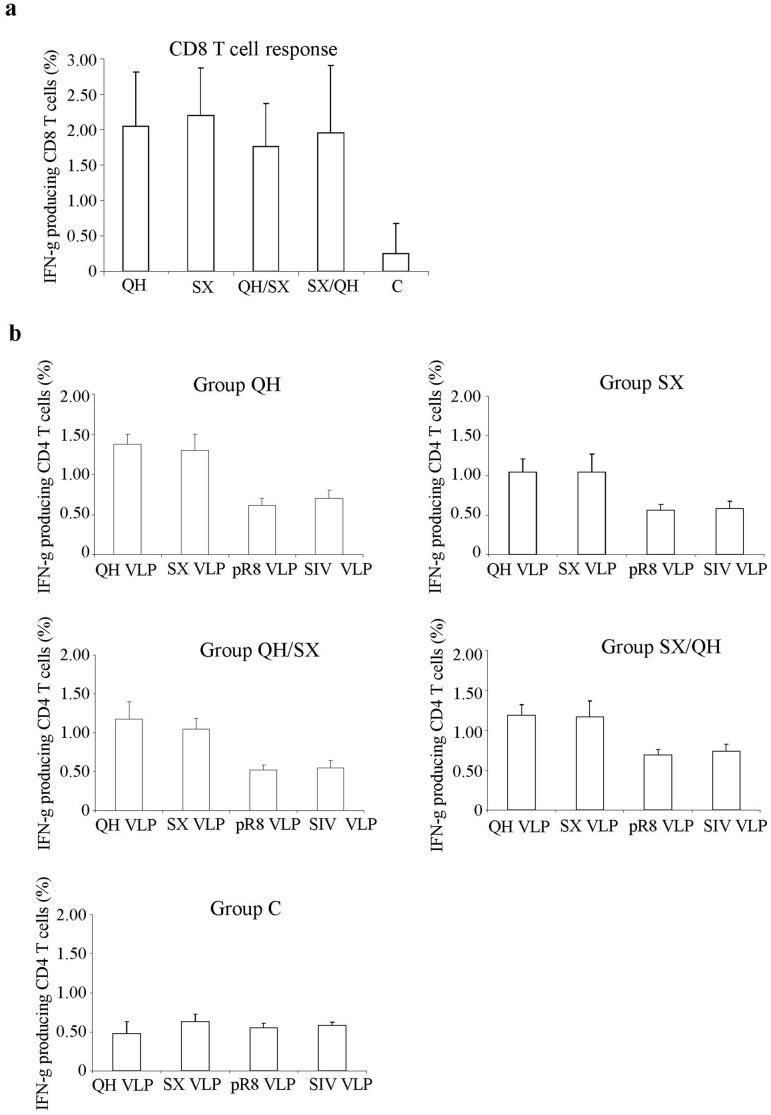
Similar levels of CD8 and CD4 T cell responses against HA were induced by different DNA immunization regimens. Mice were immunized as described in Figure 1. At two weeks after the final immunization, mice were sacrificed and splenocytes were prepared for analysis of T cell responses by intracellular cytokine staining and flow cytometry. (**a**) Comparison of CD8+ T cell responses induced in mice after DNA immunizations. Splenocytes were stimulated with a peptide corresponding to known CD8+ T cell epitopes in HA for 6 h in the presence of Brefeldin A, and then stained for cell surface CD8 as well as intracellular IFN-γ, followed by flow cytometry analysis. The percentages of IFN-γ-producing CD8+ T cells in splenocytes from each individual mouse after stimulation are shown. (**b**) Comparison of CD4+ T cell responses induced in mice after DNA immunizations. Splenocytes from immunized mice were stimulated with dendritic cells that have been pulsed with different VLPs as indicated. After 6 h stimulation in the presence of Brefeldin A, the cells were stained for cell surface CD4 as well as intracellular IFN-γ, followed by flow cytometry analysis. The percentages of IFN-γ-producing CD4+ T cells in splenocytes from each individual mouse after stimulation are shown. Data are presented as the mean ± standard deviation.

After characterization of antibody responses induced by different prime-boost immunization regimens, mice were sacrificed and splenocytes were prepared for analysis of T cell responses by intracellular cytokine staining and flow cytometry. Of note, the QH and SX HA proteins share a dominant CD8 T cell epitope for Balb/c mice (H2d), IYGRPYET (amino acid 533–541), which is also found in H1N1 human influenza A viruses. As shown in Figure 7a, immunization with the QH-HA as well as SX-HA DNA vaccines induced similar levels of CD8 T cell responses against this epitiope. The level of CD4 T cell responses induced by HA DNA vaccines were compared by stimulating mouse bone marrow derived dendritic cells (BMDC) that have been pulsed overnight with QH, SX, PR8 (A/PR/8/34) influenza virus-like particles (VLP), or SIV-Gag VLPs. As shown in Figure 7b, the levels of CD4 T cell responses against HA protein induced by immunization with HA DNA vaccines are similar in all four groups (QH, SX, QH/SX, and SX/QH) that received HA DNA vaccines. Further, the levels of IFN-gamma-producing CD4 T cells are also similar in each vaccinated group when the splenocytes were stimulated with QH or SX virus-like particle (VLP)-pulsed BMDCs. These results show that in contrast to antibody responses, the T cell responses are cross-reactive between the HA of these two H5N1 HPAIV isolates and were boosted by a heterologous HA DNA vaccine as effectively as by the homologous HA DNA vaccine. Of note, only background level of IFN-gamma-producing CD4 T cells were stimulated by BMDCs pulsed with the PR8 VLPs (A/PR/8/34) or with SIV-Gag VLPs, indicating that CD4 T cell responses induced by these H5N1 HPAIV HA DNA vaccines do not exhibit cross-reactivity to the HA of the influenza virus A/PR/8/34, which is a human influenza virus of the H1 subtype.

### Boosting but not Priming with a Heterologous HA DNA Vaccine Enhanced Protection of Mice against Lethal H5N1 Influenza Virus Challenge

**Figure 8:**
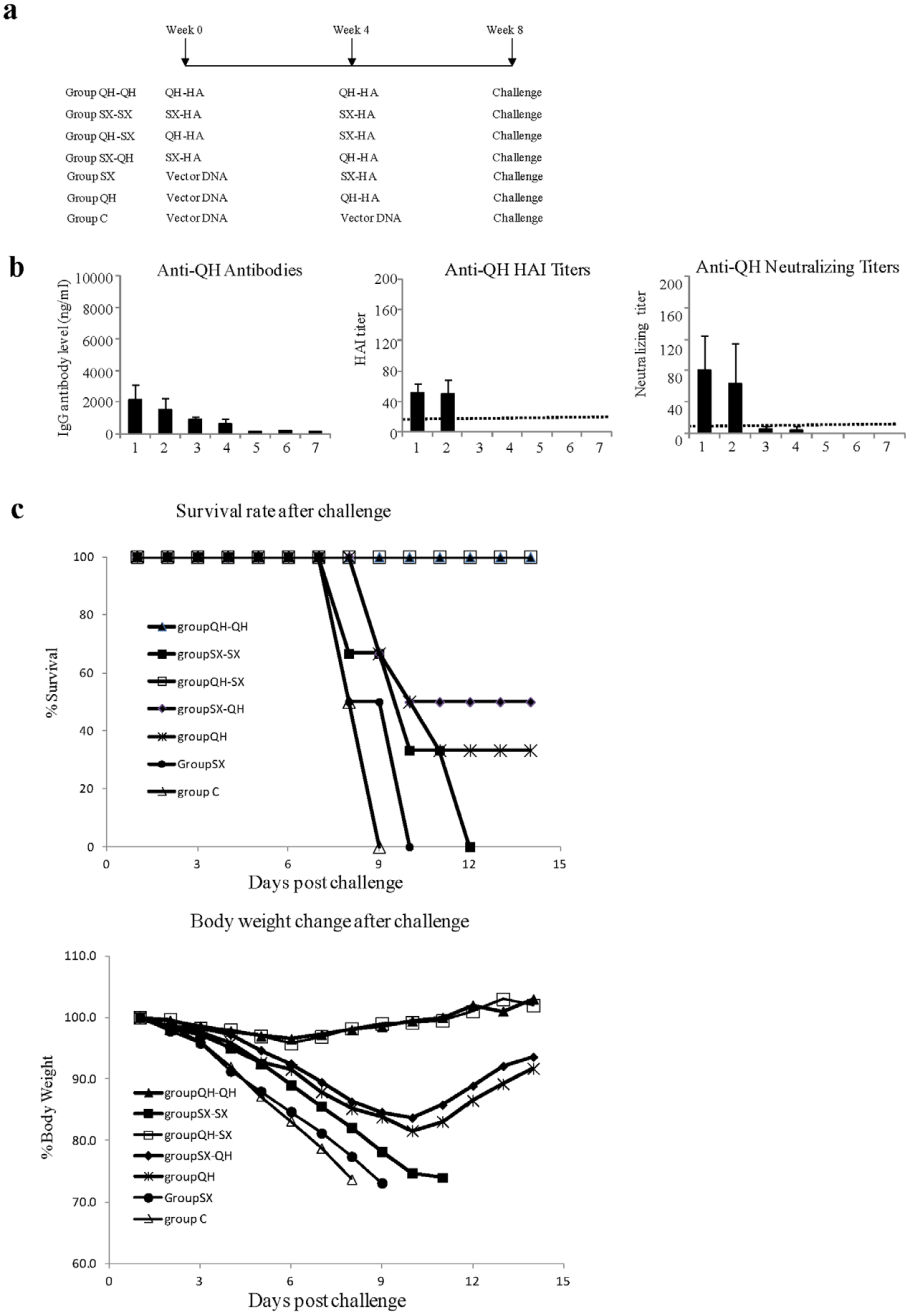
A heterologous boosting with SX HA DNA vaccine protected mice against lethal challenge by the QH H5N1 influenza virus. (**a**) Schematic diagram of the immunization and challenge studies. (**b**) At 2 weeks after the second immunization, blood samples were collected and analyzed for antibody responses against the QH virus by ELISA, HAI, and virus-neutralization assays. Data are presented as the mean ± standard deviation. Dashed lines indicate detection limit for HAI titer (1∶20) and neutralizing titer (1∶10). 1, Group QH-QH; 2, Group QH-SX; 3, Group SX-QH; 4, Group QH; 5, Group SX; 6, Group SX-SX; 7, Group C. (**c**) Mouse survival rate and body weight change after lethal influenza virus challenge. At 4 weeks after the second immunization, mice were challenged by intranasal instillation with 100 MLD50 of the QH H5N1 influenza virus and then monitored daily for weight loss and disease progression. Mice that were found to display severe signs of illness or loss more than 25% body weight were sacrificed in accordance with IACUC guidelines.

We further investigated the efficacy of different homologous and heterologous prime-boost immunization regimens for protection against challenge by the QH H5N1 HPAIV, which causes lethal infection in mice. Mice were immunized with different HA DNA vaccines as outlined in Figure 8a and then challenged with 100 MLD50 of the QH H5N1 influenza virus. Blood samples were collected after the second immunization and analyzed antibody responses against the QH virus. As shown in Figure 8b, priming with the QH HA DNA vaccine followed by boosting with the SX HA DNA vaccine induced similar levels of antibody responses against the QH virus as two immunizations with the QH HA DNA vaccine. On the other hand, priming with the SX HA DNA vaccine followed by boosting with the QH HA DNA vaccine induced similar levels of antibody responses against the QH virus as one immunization with the QH HA DNA vaccine. We further compared neutralizing activity of these sera against the QH virus. As also shown in Figure 8b, sera from the QH-SX group exhibited similar neutralizing activity against the QH virus as sera from the QH-QH group. In comparison, sera from the SX-QH group exhibited only low level neutralizing activity against the QH virus as also detected for the PBS-QH group, whereas sera from the PBS-SX and SX-SX did not show detectable neutralizing activity against the QH virus. Similar results were also obtained for HAI activity against the QH virus. These results agree with the observation in above studies, demonstrating that a heterologous boosting but not priming with the SX-HA DNA vaccine augmented antibody response against the QH virus.

The survival rates and weight changes of each group after challenge were shown in Figure 8c. The survival curves of different groups after challenge were built based on the Kaplan-Meier method and then analyzed by the Gehan-Breslow-Wilcoxon method. All mice that received two immunizations with QH HA DNA vaccine (Group QH-QH) survived the challenge by QH virus while none of the mice in the control group survived the challenge. Mice that received one (Group SX) or two (Group SX-SX) immunizations with the SX HA DNA vaccine also all succumbed to challenge, similar to the control group mice. Mice that received only a single QH HA DNA immunization (Group QH) were partially protected from the challenge, which is significantly higher compared to the control group (p<0.05). However, mice that received priming with QH HA DNA vaccine followed by boosting with SX HA DNA vaccine (Group QH-SX) were completely protected from lethal challenge by QH virus, which is significantly higher than mice that received a single immunization with QH HA DNA vaccine (p<0.05, Group QH-SX vs. Group QH). In contrast, mice that received priming with SX HA DNA vaccine followed by boosting with QH HA DNA vaccine (Group SX-QH) were only partially protected from challenge by QH virus, similar as mice that received a single immunization with QH HA DNA vaccine (p>0.05, Group SX-QH vs. Group QH). These results show that a heterologous boosting with the SX-HA DNA vaccine is as effective as two homologous QH-HA DNA immunizations for protection against a high lethal dose challenge by the QH H5N1 influenza virus, whereas a heterologous priming with the SX-HA DNA vaccine has no significant effect for protection against QH virus infection.

## Discussion

Since its first recorded outbreak in domestic poultry and humans in Hong Kong in 1997, H5N1 HPAIV has spread worldwide except in the Americas. As a result, H5N1 HPAIV has evolved into different genotypes that are antigenically distinctive from each other [16]. However, how the diversification in antigenicity of HPAIV isolates affects their immunogenicity for eliciting cross-reactive antibodies is still not clear. In this study, we characterized immune responses induced by DNA vaccines of the HA from two H5N1 HPAIV isolates, the QH and SX viruses respectively. Our results show that the antibody responses induced by this pair of HA DNA vaccines in mice are not cross-reactive, demonstrating that the HA of these two H5N1 HPAIV strains are antigenically distinctive. On the other hand, cross-reactive CD4 and CD8 T cell responses were readily induced by both HA DNA vaccines, indicating that epitopes for CD4 and CD8 T cell responses are conserved between the HA of these two viruses. Interestingly, in characterization of the antibody responses induced by a heterologous prime-boost immunization regimen, we observed that a subsequent immunization with a heterologous HA DNA vaccine selectively boosted the antibody responses against the primary virus strain but not the secondary virus strain of which the HA DNA vaccine was used in boosting immunizations. These results demonstrate for the first time the existence of OAS between two phylogenetic clades of H5N1 HPAIV that both have caused large scale outbreaks in domestic poultry and are endemic in avian species.

The rapid onset of the new H1N1 influenza virus pandemic suggests that once an influenza virus of zoonotic origin has acquired human transmissibility, it will be difficult control its spread due to the lack of herd immunity. Therefore, the preparation of a vaccine stockpile against potentially pandemic influenza viruses is of great importance. Of particular concern, outbreaks of H5N1 HPAIV over the last decade have caused over 800 human infections and different clades of H5N1 HPAIV are concurrently endemic and it is difficult to predict which strain will cause a pandemic [22]. The BHG/QH/3/05 (QH) virus represents a dominant genotype of the viruses isolated from the outbreak in wild birds in Qinghai Lake [20]. While the Qinghai-like viruses have not been detected in poultry in China after 2005, they have been repeatedly detected in wild birds or domestic poultry in other countries in Europe, Africa, and Asia [23]–[26], and were reported to be detected in pikas in the Qinghai Lake area in 2007 [27]. In addition to their wide distribution, these viruses also bear known genetic markers that are critical for transmission in mammalian hosts and thus represent a clear pandemic potential [28], [29]. The CK/SX/2/06 (SX) virus was first detected in chickens from the Shanxi province in northern China [21], which has since spread to several other provinces in northern China, including Ningxia, Henan, Shandong, and Liaoning provinces, and continues to be endemic in these regions. In addition, H5N1 HPAIV with an HA gene similar to that of CK/SX/2/06-like viruses has also been detected in chickens in Vietnam in 2008 [30]. Moreover, viruses of both clades have been reported to cause human infections [31]. Our results in this study showed that antibody responses induced by the HA of these two viruses not only failed to exert HAI activity but also exhibit almost no binding activity to each other. Further, immunization with the SX HA DNA vaccine alone conferred no protection against lethal challenge by the QH virus. The lack of cross reactivity of antibody responses between these two viruses underscores the need to prepare vaccine stockpiles for different H5N1 clades that are antigenically distinctive, to improve our preparedness against a potential H5N1 pandemic.

We further investigated whether these H5N1 HPAIV vaccines will interfere with induction of protective immune responses against an antigenically distinctive strain. Our results from this study showed that boosting immunization with a heterologous HA DNA vaccine effectively augmented antibody responses against the original vaccine strain, demonstrating the presence of OAS between these two H5N1 HPAIV strains. On the other hand, induction of antibody responses against the HA of the secondary virus strain was not affected by the immune response against the HA of the primary virus strain, differing from the OAS observed for H1N1 human influenza viruses [32]. The phenomenon of OAS was first discovered over 50 years ago from studies on human infection with different strains of H1N1 influenza A viruses [33]–[35], which was further confirmed by subsequent studies of influenza infection and vaccination in humans as well as in animal models [36]–[45]. Generally stated, OAS refers to an observation that after immunization or infection with one antigen, subsequent boosting with a second, related but heterologous antigen leads to induction of antibodies that react primarily with the first antigen rather than with the second antigen [46]. However, the underlying mechanism for the observed OAS phenomenon remains unclear. It has been suggested that heterologous boosting immunization may selectively boost memory B cells that produce cross-reactive antibodies and thus increase the level of antibody responses against the original HA antigen. At the same time, the cross-reactive memory B cells will compete with naïve B cells for the second HA antigen and thus dampen the induction of antibodies against the second HA antigen [32]. In this study, we observed that a heterologous boost augmented antibody responses against the primary strain as effectively as a homologous boost, similar as reported previously. However, we observed that induction of antibody responses against the second virus was not affected. Further, if the OAS is a result of boosting cross–reactive memory B cells, then it is expected that the antibody levels against the primary strain will be further increased after a second heterologous boost. In contrast, our results showed that a second heterologous boost significantly augmented antibody responses against the second strain but did not further increase the levels of antibody responses against the primary strain. Therefore, our results suggest that a different mechanism may be in play for the observed OAS phenomenon between these two HPAIV isolates. It is likely that the OAS between the QH and SX HA proteins is a result of reduced activation threshold of memory B cells, which are more easily stimulated by related and yet antigenically distinctive antigens as reported in early studies [47], [48]. However, when memory B cells for both HA antigens are present, activation of memory B cells for the homologous antigen exhibits a dominant effect as observed after the second heterologous boost. Taken together, these results indicate that manifestation of OAS may vary between different virus strains depending on their antigenic differences. Of note, the OAS between the QH and SX HA in this study was observed by immunization with DNA vaccines, which express the only HA antigens. In studies on H1N1 human influenza viruses, Kim et al. demonstrated OAS between influenza viruses PR8 and FM1 in both immunizations with HA DNA vaccines and sequential sublethal infections and also showed that the OAS between these two viruses is more pronounced in sequential sublethal infections [32]. Thus, it is possible that the OAS between the QH and SX H5N1 HPAIVs may also be more prominent in other vaccine platforms such as inactivated virus vaccines or virus-like particles, as these complex vaccines also share other common antigens in addition to the HA which may drive the induction of immune responses further towards the original antigen. The complexity of the OAS and its potential impact on vaccine efficacy warrant further investigation.

The OAS reported for human influenza viruses was mostly between chronologically distant strains [32], [37], [39]–[41], [44]. In these cases, the antigenic differences between these viruses are likely resulted from gradual antigenic drift over the years. Thus the OAS phenomenon may not exert a significant impact in the past for seasonal human influenza virus infection as the vaccines are regularly updated [49]. However, the 2009 H1N1 influenza pandemic introduced a new virus strain that is related to and yet antigenically different from the 2008–2009 seasonal H1N1 influenza virus. Interestingly, comparing to patterns of seasonal influenza virus infection in the past, the pandemic H1N1 influenza virus caused relatively fewer influenza-like illness in persons aged 65 or older [50]. It has been suggested that OAS may have contributed to the protection of the aged population against the new H1N1 influenza virus infection [51]. On the other hand, several reports indicated that immunization with 2008–2009 influenza vaccines may associate with a higher risk of influenza like illness caused by the new H1N1 influenza virus infection [52]–[54]. These observations raise the possibility that OAS induced by previous seasonal influenza vaccine may have enhanced infection by the new H1N1 influenza virus [52]. While these studies are not corroborated by others [55]–[59], they underscore the importance to investigate the impact of OAS on influenza vaccine efficacy and virus infection for preparation against future pandemics. Although the OAS between QH and SX viruses observed in this study did not affect antibody induction to the second strain, it remains to be determined whether a different outcome will be found between H5N1 HPAIV from other clades. Further, while the HA of QH and SX viruses antigenically distinctive, both CD8 and CD4 T cell responses induced by these two HA DNA vaccines are cross reactive and T cell responses were effectively augmented by heterologous boosting similar as homologous boosting. Evidence suggests that cross-reactive T cell responses may also contribute to protection against influenza virus infection [60]. However, while it is expected that priming with the SX HA DNA followed by boosting with the QH HA DNA will elicit higher levels of CD8 and CD4 T cell responses than a single immunization with the QH HA DNA vaccine, such enhanced T cell response apparently did not result in improved protection against subsequent challenge by the QH H5N1 virus. Thus, such cross reactive CD4 and CD8 T cell responses may not be very effective for protection against infection and pathogenesis by H5N1 HPAIV. Moreover, the impact of OAS to protection against subsequent heterologous H5N1 HPAIV infection in humans and other animals may also exhibit individual differences. As reported in our recent studies, evolution of H5N1 HPAIV has resulted in continuing antigenic drift of these viruses [19]. The genetic and biological complexity of the H5N1 HPAIV detected in recent years in China alone indicates that reassortant viruses are constantly generated during natural infection of avian species, which may enable these lethal viruses to gain transmissibility in humans. In particular, the OAS observed in this study between two antigenically distinctive HA antigens did not significantly affect the induction of antibody responses against the second virus strain. However, evolution of H5N1 HPAIV may lead to emerge of viruses with antigenic-drifted HAs that share non-neutralizing epitopes, which could potentially exert a more deleterious effect on induction of protective immune responses against other strains. Therefore, the existence of OAS between different clades of H5N1 HPAIV is also of significant concern for H5N1 influenza vaccine development. In a recent study, Want et al. showed that immunization with polyvalent DNA vaccines successfully induced cross-protective immune responses against several H5N1 HPAIVs from different clades [61]. Further, Giles et al. reported the design of a computationally-optimized HA that could confer cross-protection against infection by different strains of HPAIV [62]. These studies offer promising strategies to overcome the potential impact of OAS between different HPAIVs. The rapid evolution of antigenically diverse H5N1 HPAIV in domestic poultry and wild birds underscores the importance of surveillance studies to determine cross-reactivity between different virus strains and the need to develop an effective polyvalent or universal vaccine strategy against a potential H5N1 influenza pandemic.

## Materials and Methods

### Ethics Statement

Animal ethics approval for the immunization studies in mice and guinea pigs was obtained from the Institutional Animal Care and Use Committee (IACUC) at the Emory University. All animal studies were performed in compliance with the guidelines of the Institutional Animal Care and Use Committee (IACUC) under approval from the IACUC at the Emory University.

### Production of DNA Vaccines

The genes for the HA of QH and SX H5N1 viruses were amplified by RT-PCR following established protocols and then cloned into the plasmid vector pCAGGS (kindly provided by Dr. Kowaoka) under the chicken beta-actin promoter. Plasmids were amplified in *E. coli* DH5α and purified with an Endo-Free Megaprep DNA purification kit (Qiagen) following manufacturer’s protocols. The plasmids were then resuspended at 1 µg/µl in sterile PBS and stored at –80°C until being used for immunizations. Expression of HA was characterized by transfection of HeLa cells using Lipofectamine2000 (Invitrogen) followed by SDS-PAGE and Western blot using mouse immune sera against QH and SX HA.

### Immunization and Blood Sample Collection

Female BALB/c mice (6–8 weeks old) were obtained from Charles River Laboratory (Charleston, SC) and housed at the Emory University animal facility in micro-isolator cages. For DNA immunization, 50 µg of HA DNA vaccines were dissolved in 100 µl PBS and then injected into both side mouse quadriceps (50 µl per side). At two weeks after each immunization, blood samples were collected by retro-orbital bleeding, heat-inactivated, and stored at −80°C until further analysis.

### ELISA

Influenza HA-specific antibodies were measured in individual mouse sera by an ELISA following established protocols ([63], [64]). Recombinant influenza viruses containing the HA and NA of QH and SX H5N1 isolates and internal genes from PR8 influenza virus (6+2) were rescued by reverse genetics, which were grown in SPF chicken embryonated eggs, inactivated by formalin treatment, and purified by centrifugation. The purified inactivated virus was then used as coating antigens in ELISA for detection of HA specific antibodies. A standard curve was constructed by coating each ELISA plate with serial 2-fold dilutions of purified mouse IgG with known concentration. The concentrations of influenza HA-specific antibodies in serum samples were calculated using the obtained standard curves and expressed as the amount of HA-specific antibody in 1 ml of serum samples (ng/ml).

### Hemagglutination Inhibition (HAI) Assay

The HAI assay was performed as described previously ([64], [65]). Briefly, mouse sera were heat-inactivated at 56°C for 1 h and then treated with receptor destroying enzyme (Denka Seiken, Tokyo, Japan) at 37°C overnight according to the manufacturer’s instruction. After treatment, 25-µl aliquots of 2-fold serially diluted serum samples were added to 25-µl PBS containing 4 HA units of purified inactivated recombinant influenza virus, After incubation at 37°C for 1 h, the serum-virus mixture was then incubated with 50 µl of 0.5% chicken red blood cells (LAMPIRE Biological Laboratories, Pipersville, PA) at 25°C for 45 min in a u-bottom 96-well plate. The HAI titer was defined as the reciprocal of the highest serum dilution that inhibited hemagglutination.

### Intracellular Cytokine Staining and Flow Cytometry

Mice were sacrificed on day 14 after the final immunization to prepare splenocytes for analysis of T cell responses by intracellular cytokine staining coupled with flow cytometry following established protocols ([66], [67]). Splenocytes were stimulated with a peptide corresponding to a CD8+ T cell epitope for the influenza virus HA (IYSTVASSL, synthesized at the Emory Microchemical Facility, Atlanta, GA) or an irrelevant peptide corresponding to a segment in the HIV Gag protein (AMQMLKETI, negative control) at 10 µg/ml for 6 h in presence of 10 µg/ml Brefeldin A (Sigma, St. Louis, MO), and CD8+ T cell responses were determined by intracellular cytokine staining of IFN-gamma and analyzed by flow cytometry on a BD FACSCalibre with CELLQuest software (Becton Dickinson, Franklin Lakes, NJ). For detection of HA-specific CD4+ T cells, bone marrow-derived dendritic cells (BMDC) were prepared following established procedures ([63]), incubated with influenza virus virus-like particles containing the HA of QH or SX (10 µg/ml) overnight, and then mixed with mouse splenocytes at 1∶5 ratio for 6 h in presence of 10 µg/ml Brefeldin A. The levels of CD4+ T cell responses were determined by intracellular cytokine staining of IFN-gamma and analyzed by flow cytometry on a BD FACSCalibre with CELLQuest software.

### Challenge Studies

Challenge of mice with H5N1 HPAIV was carried out in a biosafety-level 3 animal facility at the Harbin Veterinary Research Institute in China, and all challenged mice were housed in high-efficiency particulate air-filtered (HEPA-filtered) isolators. Groups of eight 6- to 8-week-old female BALB/c mice (Beijing Experimental Animal Center, Beijing) were immunized by different DNA vaccine regimens. The wild type QH H5N1 influenza virus (A/bar-headed goose/Qinghai/3/2005) was grown in 10-day old SPF (special pathogen free) chicken embryonated eggs and stored in −80°C until use. Virus was titered in 10-day old SPF chicken embryonated eggs to determine EID50 (egg-infectious dose) and MLD50 (mouse Lethal Dose) was determined in 8-week old female Balb/c mice. At 4 weeks after the final immunization, mice were lightly anesthetized with CO_2_ and inoculated intranasally with 100 MLD50 of QH H5N1 influenza virus (which is approximately 1000 EID50) in a volume of 50 µl and monitored daily for weight loss and mortality.

### Virus Neutralization Assay

Sera were heat-inactivated at 56°C for 1 h, and then mixed with 100 TCID50 (tissue culture infectious dose) of the QH H5N1 influenza virus in serial 2-fold dilutions (starting dilution 1∶10). After 1 h incubation, the virus-sera mixtures were added to MDCK cells that were seeded in a 96-well plate for 1 h (duplicate in 6 wells for each sample dilution). The cells were then replaced with complete media. At 48 h post infection, medium was collected and analyzed for hemagglutination activity (HA). The virus-neutralizing titer was defined as the reciprocal of the highest serum dilution that inhibited hemagglutination activity by medium from infected cells.

### Statistical Analysis

The average value and standard deviation for the level of immune responses within each group were calculated for comparison and the significance of the differences between the results from different groups was determined by a *student t test* using the Excel program (Microsoft, Redmond, WA). The statistical analysis of the survival curves were carried out using the GraphPad Prism (La Jolla, CA) software. The survival curves of different groups after challenge were built based on the Kaplan-Meier method and post-test comparison of the curves of two different groups was analyzed by the Gehan-Breslow-Wilcoxon method.

## Supporting Information

Figure S1 **Characterization of QH and SX HA expression by DNA vaccines.** HeLa cells were grown to confluence in a six-well plate and then transfected by QH or SX HA DNA vaccines using Lipofectamine2000. At 24 hr post transfection, cell lysate was analyzed by SDS-PAGE and Western blot. Expression of the HA proteins was detected using a mixture of mouse sera against QH and SX HA as primary antibodies and HRP-conjugated goat-anti-mouse antibodies as secondary antibodies. Lane C, Control transfection by DNA vector pCAGGS; lane QH, transfection by QH HA DNA vaccine; lane SX, transfection by SX HA DNA vaccine.
(TIFF)

Table S1 **Summary of antibody responses against QH and SX viruses after each immunization. a.** Antibody levels against QH and SX viruses after each immunization**. b.** HAI titers against QH and SX viruses after each immunization**.**
(DOC)
