# Supplementary figures and images for: Characterization of Immune Responses Induced by Immunization with the HA DNA Vaccines of Two Antigenically Distinctive H5N1 HPAIV Isolates
Source: PLoS One. 2012 Jul 31;7(7):e41332. doi: 10.1371/journal.pone.0041332 (PMC3409192; doi:10.1371/journal.pone.0041332)

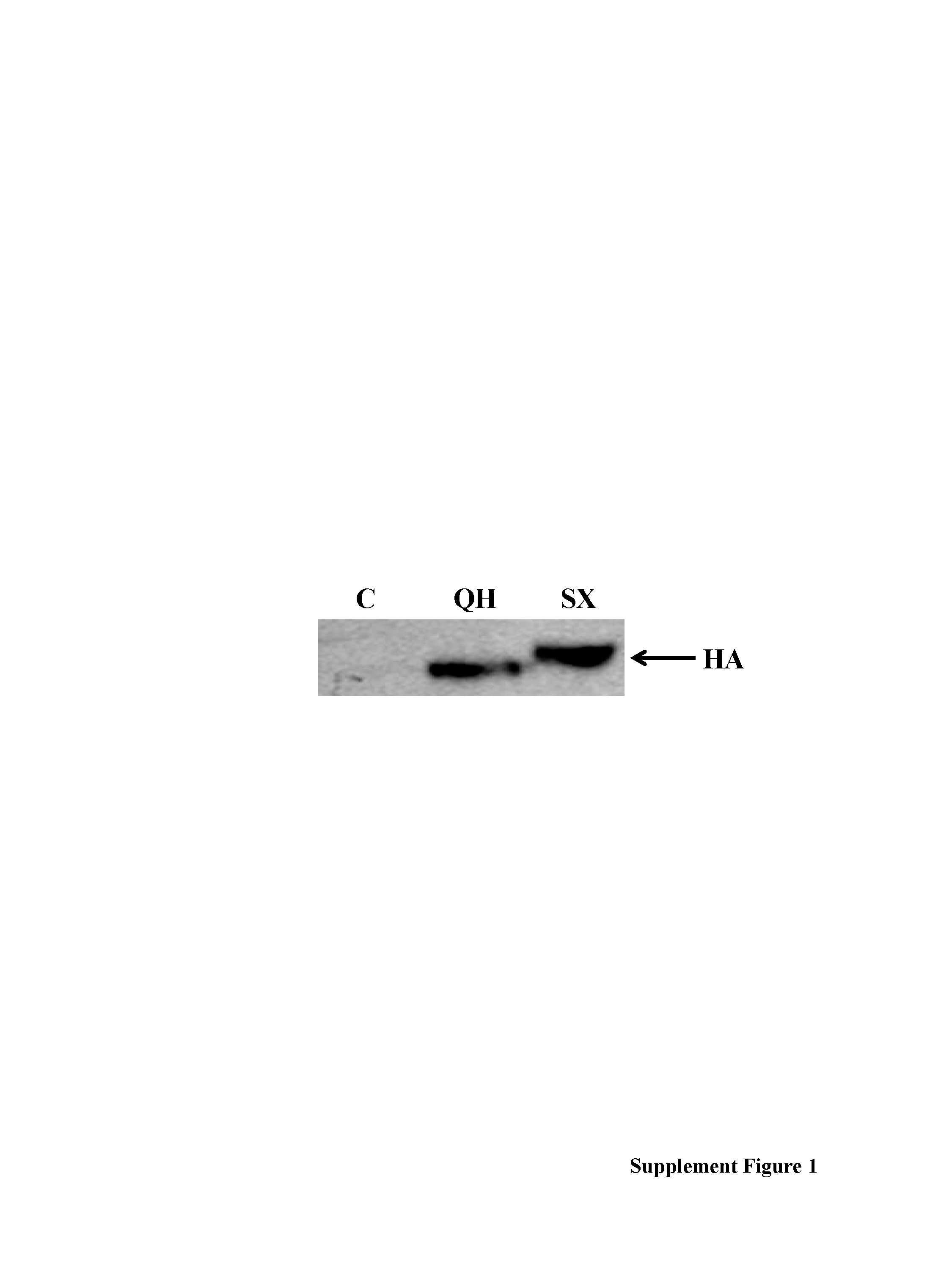

Supplement: Figure S1 — Characterization of QH and SX HA expression by DNA vaccines. HeLa cells were grown to confluence in a six-well plate and then transfected by QH or SX HA DNA vaccines using Lipofectamine2000. At 24 hr post transfection, cell lysate was analyzed by SDS-PAGE and Western blot. Expression of the HA proteins was detected using a mixture of mouse sera against QH and SX HA as primary antibodies and HRP-conjugated goat-anti-mouse antibodies as secondary antibodies. Lane C, Control transfection by DNA vector pCAGGS; lane QH, transfection by QH HA DNA vaccine; lane SX, transfection by SX HA DNA vaccine. (TIFF) [file pone.0041332.s001.tiff]
