# Supplementary material for: Characterization of Immune Responses Induced by Immunization with the HA DNA Vaccines of Two Antigenically Distinctive H5N1 HPAIV Isolates
Source: PLoS One. 2012 Jul 31;7(7):e41332. doi: 10.1371/journal.pone.0041332 (PMC3409192; doi:10.1371/journal.pone.0041332)
Supplement: Table S1 — Summary of antibody responses against QH and SX viruses after each immunization. a. Antibody levels against QH and SX viruses after each immunization. b. HAI titers against QH and SX viruses after each immunization. (DOC) [file pone.0041332.s002.doc]

**Table S1.** Summary of antibody responses against QH and SX viruses after each immunization

**a.** Antibody levels against QH and SX viruses after each immunization

|  | Antibody level against QH virus | | | | Antibody level against SX virus | | | |
| --- | --- | --- | --- | --- | --- | --- | --- | --- |
| Group | 1st immunization | 2nd immunization | 3rd immunization | 4th immunization | 1st immunization | 2nd immunization | 3rd immunization | 4th immunization |
| QH | 837 | 3606 | 6015 | 7941 | 186 | 163 | 197 | 296 |
| SX | 83 | 349 | 823 | 858 | 272 | 1011 | 2769 | 3236 |
| QH/SX | 857 | 3221 | 6168 | 5513 | 83 | 233 | 314 | 1197 |
| SX/QH | 230 | 377 | 887 | 4248 | 255 | 832 | 2294 | 2215 |
| C | 201 | 189 | 235 | 210 | 181 | 169 | 205 | 190 |

Note: Antibody levels are expressed as the calculated concentration of virus-specific antibodies in serum samples (ng/ml).

**b.** HAI titers against QH and SX viruses after each immunization

|  | HAI titer against QH virus | | | | HAI titer against SX virus | | | |
| --- | --- | --- | --- | --- | --- | --- | --- | --- |
| Group | 1st immunization | 2nd immunization | 3rd immunization | 4th immunization | 1st immunization | 2nd immunization | 3rd immunization | 4th immunization |
| QH | <20 | 40 | 100 | 120 | <20 | <20 | <20 | <20 |
| SX | <20 | <20 | <20 | <20 | <20 | 40 | 120 | 120 |
| QH/SX | <20 | 40 | 100 | 40 | <20 | <20 | <20 | 50 |
| SX/QH | <20 | <20 | <20 | 50 | <20 | 60 | 100 | 80 |
| C | <20 | <20 | <20 | <20 | <20 | <20 | <20 | <20 |

Note: Detection limit for HAI titers is 1:20.
